# An Investigation of the Photonic Application of TeO_2_-K_2_TeO_3_-Nb_2_O_5_-BaF_2_ Glass Co-Doped with Er_2_O_3_/Ho_2_O_3_ and Er_2_O_3_/Yb_2_O_3_ at 1.54 μm Based on Its Thermal and Luminescence Properties

**DOI:** 10.3390/ma17174188

**Published:** 2024-08-23

**Authors:** Ahlem Boussetta, Aref M. Al-Syadi, Hasan B. Albargi, Kamel Damak, Ali Erçin Ersundu, Miray Çelikbilek Ersundu, Essam Ramadan, Ali M. Alshehri, Khalid I. Hussein, Ramzi Maalej, El Sayed Yousef

**Affiliations:** 1 Laboratory of Systems Integration and Emerging Energies, National Engineering School of Sfax (ENIS), University of Sfax, Sfax 3018, Tunisia; ahlem.bousseta@ipeis.usf.tn; 2 Department of Physics, Faculty of Science and Arts, Najran University, Najran 11001, Saudi Arabia; arefalsyadi@yahoo.com (A.M.A.-S.); hbalbargi@nu.edu.sa (H.B.A.); 3 Promising Centre for Sensors and Electronic Devices (PCSED), Advanced Materials and Nano-Research, Najran University, Najran 11001, Saudi Arabia; 4 LaMaCoP, Faculty of Sciences of Sfax, University of Sfax, Sfax 3018, Tunisia; kamel.damak@ipeis.usf.tn (K.D.); ramzi.maalej@fss.usf.tn (R.M.); 5 Department of Metallurgical and Materials Engineering, Yildiz Technical University, Davutpasa Campus KMA-205, Esenler, Istanbul 34220, Turkey; ersundu@yildiz.edu.tr (A.E.E.); miray@yildiz.edu.tr (M.Ç.E.); 6 Physics Department, Faculty of Science, Al Azhar University, Assiut 71542, Egypt; esam_ramadan2008@yahoo.com; 7 Department of Physics, Faculty of Science, King Khalid University, Abha 61421, Saudi Arabia; amshehri@kku.edu.sa (A.M.A.); ayousf@kku.edu.sa (E.S.Y.); 8 Department of Radiological Sciences, College of Applied Medical Sciences, King Khalid University, Abha 61421, Saudi Arabia

**Keywords:** co-doping tellurite glass, rare earth, DSC, thermal characteristics, linear and nonlinear refractive index, Judd–Ofelt analysis

## Abstract

A glass composition using TeO_2_-K_2_TeO_3_-Nb_2_O_5_-BaF_2_ co-doped with Er_2_O_3_/Ho_2_O_3_ and Er_2_O_3_/Yb_2_O_3_ was successfully fabricated. Its thermal stability and physical parameters were studied, and luminescence spectroscopy of the fabricated glasses was conducted. The optical band gap, *E*_opt_, decreased from 2.689 to 2.663 eV following the substitution of Ho_2_O_3_ with Yb_2_O_3_. The values of the refractive index, third-order nonlinear optical susceptibility (χ^(3)^), and nonlinear refractive index (n_2_) of the fabricated glasses were estimated. Furthermore, the Judd–Ofelt intensity parameters $\Omega_{t}\;(t=2,4,6)$, radiative properties such as transition probabilities (*A_ed_*), magnetic dipole-type transition probabilities (*A_md_*), branching ratios (*β*), and radiative lifetime (τ) of the fabricated glasses were evaluated. The emission cross-section and FWHM of the ^4^*I*_13/2_→^4^*I*_15/2_ transition around 1.54 μm of the glass were reported, and the emission intensity of the visible signal was studied under 980 nm laser excitation. The material might be a useful candidate for solid lasers and nonlinear amplifier devices, especially in the communications bands.

## 1. Introduction

Due to their unique optical characteristics, glasses are frequently utilized as optical materials. In recent years, there has been an obvious increase in interest in the possible applications of tellurite glasses due to their intriguing and significant optical features. Tellurite glasses have been used to fabricate a wide range of devices, such as planar waveguides, nanowires, and optic amplifiers [1,2,3,4]. Numerous studies have been conducted to examine the physical characteristics of several tellurite (TeO_2_) glass combinations. TeO_2_ glasses have attracted some technical and scientific interest because of their many applications [5]. Technological optical fiber devices, lasers, optical fibers, solar cells, sensors, memory-switching devices, gas sensors, optoelectronics, and optical waveguide applications have all demonstrated significant potential for using these glasses [5,6,7,8]. Furthermore, due to TeO_2’_s excellent nonlinear optical characteristics, low melting point, superior chemical stability, and elevated index of refraction, it has drawn greater interest as a glass former than other glass formers such as silicates and phosphate [9,10,11]. Given their huge transparent window, great refractive index, and outstanding stability, TeO_2_ glasses containing a heavy metal oxide—such as Nb_2_O_5_, which is a heavy metal tellurite glass—are appealing for the additional development of infrared lasers and amplifiers [12]. Furthermore, niobic TeO_2_ glasses have an elevated third-order nonlinear optical susceptibility, which makes them a viable option for nonlinear fiber devices such as all-optical switches [12]. The contents of non-bridging oxygen (NBO) and TeO_3_ units both rise when modifiers such as BaO_2_ disrupt the random arrangement of glasses [13]. Moreover, the alkaline earth metal element barium (Ba) has a high basicity, a large ionic radius, and a higher atomic number. Consequently, the presence of Ba in the TeO_2_ glass framework modifies the glass’s construction and enhances its chemical stability, density, gloss, and refractive index [14,15]. When fluorine ions are added to TeO_2_ glass, the glass’s formation range is increased, its viscosity is decreased, its degree of transparency is enhanced, and its moisture resistance is increased [16]. Also, adding fluorine to TeO_2_ glasses disintegrates the network of the glassy system due to fluorine’s electronegativity being greater than that of oxygen [16]. This implies that the network’s configuration of the glass in these kinds of glasses is impacted by the fluoride’s replacement of oxygen. When the framework is altered, a lot of fundamental characteristics also change. Unlike both oxygen and fluorine matrix structures, an oxide–fluoride glass matrix might offer rare earth ions a unique home. As a result, oxyfluoride glasses with a high proportion of rare earth elements are novel beneficial substances [17]. Fluoride glasses have a minimal cost limit and a wide spectrum range, which makes them ideal for optical fiber applications involving sensors. It is commonly known that increasing a glass matrix’s metal fluoride compounds improves its transparent nature and refractive index [9]. The prospective utilization of co-doping of glasses with rare earth ions in solid-state lasers, three-dimensional displays, and optical amplifiers has garnered significant interest in the last decade [18]. The rare earth ions erbium (Er^3+^), ytterbium (Yb^3+^), and holmium (Ho^3+^) have received the most attention. Er^3+^, Ho^3+^, and Yb^3+^ ion insertion into the matrix of glass allows for the production of up-conversion luminescence when it is exposed to mid-infrared (MIR) rays. A high-power laser diode energy of 980 nm might be used to actively excite Er^3+^ ions. According to the mutual concentration of these ions, a transfer of energy process among them might then alter the up-conversion emission intensity [19]. Moreover, erbium-doped glasses are extensively utilized in a diversity of optical implementations, mostly in the areas of eye-safe lasers and optical amplifiers for fiber networking [18,20,21]. Furthermore, the observable up-conversion emission can be amplified by co-doping the host substance with Ho_2_O_3_/Er_2_O_3_ or Yb_2_O_3_/Er_2_O_3_ couplings. This is because of a greater absorbing cross-section and greater energy transmission operations from Ho^3+^ to Er^3+^ ions or from Yb^3+^ to Er^3+^ ions, respectively [18,22,23]. The present work aimed to prepare TeO_2_-based glass (70TeO_2_-15K_2_TeO_3_-10Nb_2_O_5_-5BaF_2_) that was co-doped with Er_2_O_3_/Ho_2_O_3_ and Er_2_O_3_/Yb_2_O_3_ and to investigate the impact of these rare earth oxides on the thermal and optical properties of the glasses. Differential scanning calorimetry and a double-beam spectrophotometer were utilized to study these characteristics. Furthermore, optical spectrum measurements were conducted to estimate the optical factors. The energy levels of the glass co-doped with Er_2_O_3_/Yb_2_O_3_ were determined, and the branching ratios (*β*), radiative lifetimes (*τ*_rad_), electric dipole-type transition probabilities (*A*_ed_), magnetic dipole-type transition probabilities (*A*_md_), and Judd–Ofelt intensity factors *Ω*_t_ (t = 2, 4, 6) were calculated.

The primary goal of this work was to investigate how the amount of Yb^3+^ and Ho^3+^ affects the spectroscopic characteristics of Er^3+^-co-doped TeO_2_ glasses. This will help maximize the transition’s gain and emission cross-section between ^4^*I*_13/2_→^4^*I*_15/2_ and will also determine whether or not these glasses are suitable as optical glasses for laser and fiber amplifiers. Utilizing measurements of the absorption spectra and McCumber theory, the absorbing, emitting, and gain cross-sections of the ^4^*I*_13/2_→^4^*I*_15/2_ transition were derived at approximately 1.54 µm. Finally, the emission intensity of the visible signal was studied under 980 nm laser excitation. The FWHM of the ^4^*I*_13/2_→^4^*I*_15/2_ transition of the glass was reported. Furthermore, we estimated the nonlinear refractive index and third-order susceptibility of fabricated glass which have good transmission with multiple absorption peaks in the near-infrared wavelength spectrum. Therefore, fabricated glass is a unique property that can used in nonlinear devices.

## 2. Experimental Section

The TeO_2_ glasses with the composition 70TeO_2_-15K_2_TeO_3_-10Nb_2_O_5_-5BaF_2_ (in mol%) co-doped with Er_2_O_3_/Ho_2_O_3_ and Er_2_O_3_/Yb_2_O_3_ were produced using a melt-quenching process. All the starting chemicals were procured from Aldrich and were 99.99% pure. The particulars of their compositions are displayed in Table 1.

**Figure 1:**
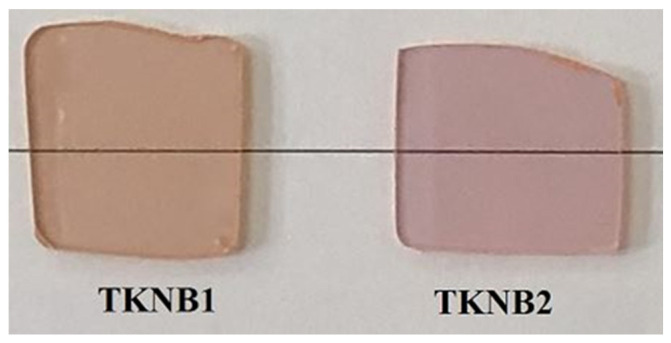
Photo of polished glass samples of 70TeO_2_-15K_2_TeO_3_-10Nb_2_O_5_-5BaF_2_-30,000 ppmHo_2_O_3_-30,000 ppm Er_2_O_3_ (TKNB1) and 70TeO_2_-15K_2_TeO_3_-10Nb_2_O_5_-5BaF_2_-30,000 ppmYb_2_O_3_-30,000 ppm Er_2_O_3_ (TKNB2).

After mixing, the mixture was heated for 25 min at 950 °C in a platinum crucible within a furnace. After that, a graphite mold was filled with the extremely viscous melt. Following two hours of annealing at 270 °C, the quenched glass was gradually cooled to room temperature (RT). Figure 1 shows pictures of the sample glasses as they were produced. The samples were cut and polished to 2.1 mm thick. To investigate the thermal properties of these glasses, a DSC Shimadzu 50 with a resolution ± 1.0 °C at a heating rate of 10 °C/min over a temperature range of 550 °C was employed. Toluene, a known-density immersing solution (0.8669 g/cm^3^), was employed using the Archimedes method with a resolution ± 0.001 g/cm^3^ to determine the glass sample’s density at RT. In the 190–2500 nm wavelength range with a resolution of 1nm, the optical absorbing and transmission spectra were attained utilizing a JASCO V-570 spectrophotometer.

## 3. Results and Discussion

### 3.1. Thermal Characteristics

**Figure 2:**
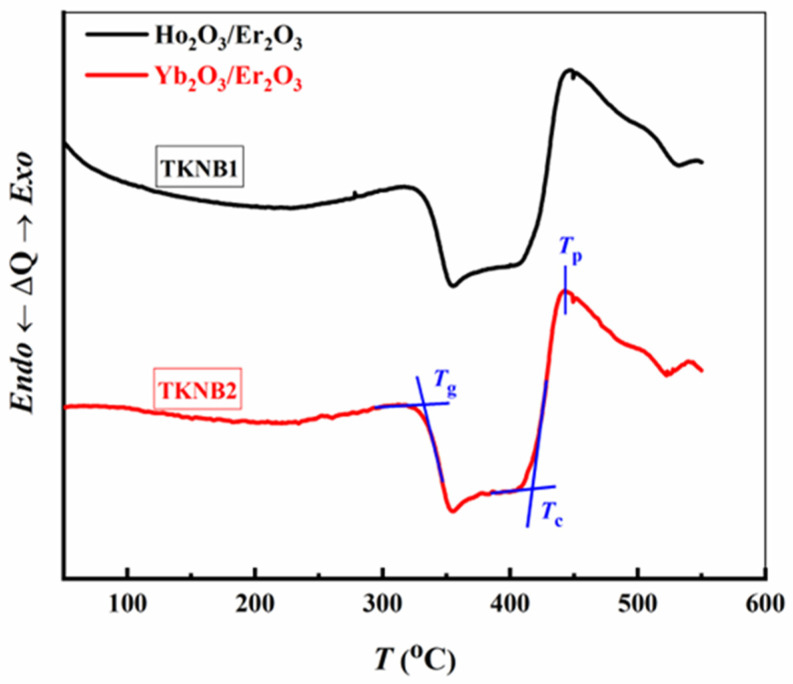
DSC thermograms for TKNB glass doped with Ho_2_O_3_/Er_2_O_3_ and Yb_2_O_3_/Er_2_O_3_.

The DSC thermograms for the 70TeO_2_-15K_2_TeO_3_-10Nb_2_O_5_-5BaF_2_ glass co-doped with Er_2_O_3_/Ho_2_O_3_ and Er_2_O_3_/Yb_2_O_3_ at a rate of 10 °C/min are shown in Figure 2. These graphs demonstrate that at the glass transition, distinct endothermic peaks are seen, followed by an exothermic crystallization peak. Table 2 lists the glass transition temperature (*T*_g_), crystallization start temperature (*T*_c_), and peak crystallization temperature (*T*_p_) for the investigated glasses.

The glassy nature of the current samples was confirmed based on the curve forms. Additionally, *T*_g_ offers details regarding the glass network’s interconnectivity and binding strength. It is understood that *T*_g_ increases as the glass’s interconnectivity and binding strength grow [24]. These values of (*T*_g_) are close to the tellurite-based glasses [25]. The distinction between *T*_c_ and *T*_g_ is employed to determine the thermal stability (Δ*T*) [26,27] of the produced glasses (Δ*T* = *T*_c_ − *T*_g_). The glass’s excellent thermal stability is indicated by the significant difference between *T*_c_ and *T*_g_. Another method for calculating the glass’s stability would be to use Sestak’s estimation of the Hruby index, *H* = Δ*T*/*T*_g_ [28,29]. The ∆*T* and *H* are shown in Table 2, and these are important for determining the glass devitrification process [27]. The next relation could be employed to evaluate the value of the factor *K*_SP_, which is associated with glass’s stability versus crystallization [28,29,30,31]:

(1) $$K_{SP}=\frac{\left(T_{p}-T_{c}\right)\left(T_{p}-T_{g}\right)}{T_{g}}$$

The *K*_SP_ magnitudes of the produced glasses are documented in Table 2. It can be seen that all these values of the thermal stability parameters (∆*T*, *H*, *K*_SP_) of the TKNB glass co-doped with Er_2_O_3_/Yb_2_O_3_ (TKNB2) are slightly lower than those of the TKNB glass co-doped with Er_2_O_3_/Ho_2_O_3_ (TKNB1). This may suggest a decrease in rigidity in the glassy matrix due to the replacement of Ho_2_O_3_ with Yb_2_O_3_. On the other hand, this decrease in thermal stability due to the substitution of Ho_2_O_3_ with Yb_2_O_3_ might be credited to the following causes: (i) the low bond strengthening Yb-O (387.7 kJ mol^−1^) in contrast to Ho-O (606 kJ mol^−1^) [32]; or (ii) the cation radius of Yb^3+^ (2.28 Å) being slightly lower than the cation radius of Ho^3+^ (2.33 Å). In addition to reducing the length of bonds and producing a high coulombic force of attraction between opposing ions, cation’s radius is directly proportional to polarizability [33].

### 3.2. Density and Molar Volume

The densities of the examined glasses were determined by utilizing Archimedes’ principle. We used toluene (*ρ*_0_ = 0.8669 g/cm^3^) as the immersing fluid. The density (*ρ*) was computed employing the equation presented below after the weights of the glasses were first measured separately in the air (*W*_a_) and then in the previously mentioned liquid (*W*_l_).

(2) $$\rho =\frac{W_{a}}{W_{a}-W_{l}}\rho_{0}$$

Using the formula *V*_m_ = *M*_w_/*ρ*, where *M*_w_ is the glass sample’s molecular weight, the molar volume (*V*_m_) was computed. The values of *ρ* and *V*_m_ are listed in Table 1. It is worth mentioning that the substitution of Ho_2_O_3_ with Yb_2_O_3_ leads to a slight increase in glass density, while the *V*_m_ slightly decreases. This phenomenon could be ascribed to both the higher *ρ* and the higher *M*_w_ of Yb_2_O_3_ (9.17 g/cm^3^, 394.08 g/mol) compared with Ho_2_O_3_ (8.41 g/cm^3^, 377.86 g/mol). Given that the *ρ* is inversely correlated to the *V*_m_ and is proportionate to the average *M*_w_, it is generally predicted that the two quantities will behave in opposition to one another.

### 3.3. Optical Properties

**Figure 3:**
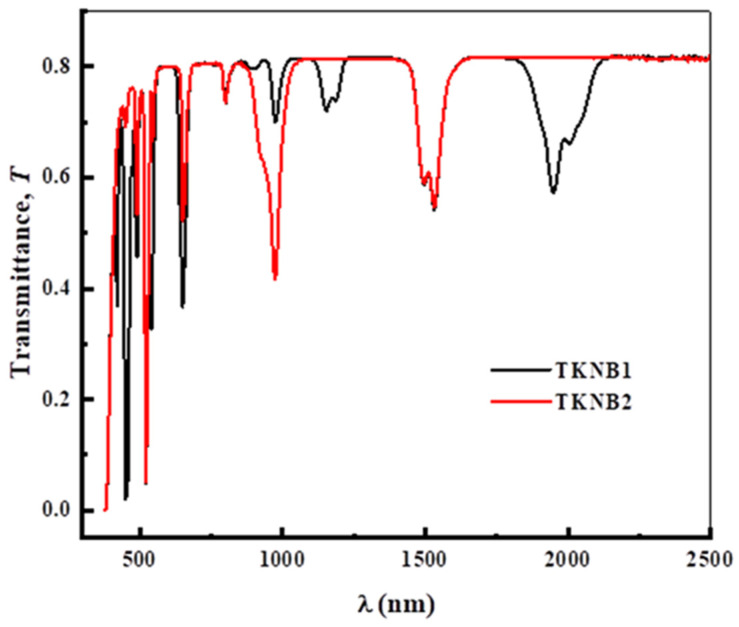
Optical transmission spectra of the TKNB glass doped with Ho_2_O_3_/Er_2_O_3_ and Yb_2_O_3_/Er_2_O_3_.

**Figure 4:**
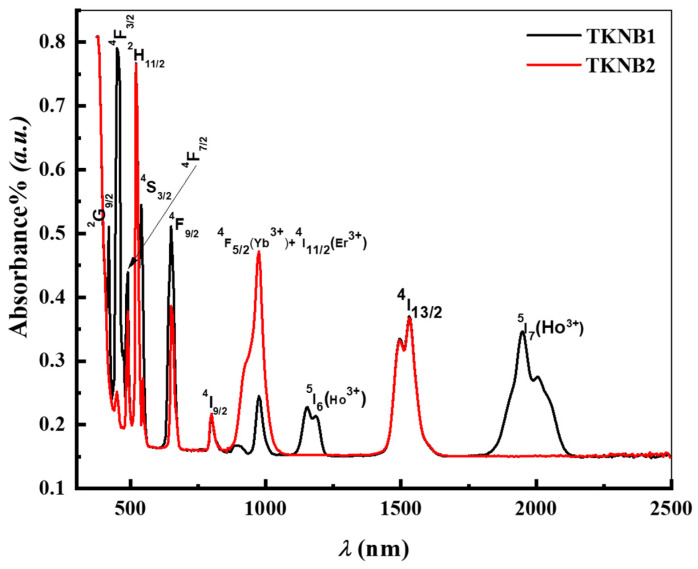
Optical absorption spectra of the TKNB glass doped with Ho_2_O_3_/Er_2_O_3_ and Yb_2_O_3_/Er_2_O_3_.

The spectra of optical transmission of the TKNB glasses co-doped with Er_2_O_3_/Ho_2_O_3_ and Er_2_O_3_/Yb_2_O_3_ are provided in Figure 3, which indicates that the fabricated glasses are highly transmissible in the range of UV–VIS–NIR wavelengths. Figure 4 demonstrates several peaks in the spectra, which are due to the presence of Er^3+^, Ho^3+^, and Yb^3+^ ions in the TKNB glass system co-doped with Er_2_O_3_/Ho_2_O_3_ and Er_2_O_3_/Yb_2_O_3_. The absorption bands corresponding to the transition from ^4^*I*_15/2_ to the ground states ^5^*I*_7_(Ho^3+^), ^4^*I*_13/2_, ^5^*I*_6_(Ho^3+^), ^4^*I*_11/2_(Er^3+^) + ^4^*F*_5/2_(Yb^3+^), ^4^*I*_9/2_, ^4^*F*_3/2_, ^4^*S*_3/2_, ^2^*H*_11/2_, ^4^*F*_7/2_, ^4^*F*_3/2_, and ^2^*G*_9/2_ can be attributed to the following wavelengths: 1950, 1534, 1155, 970, 803, 655, 540, 520, 490, 450, and 420 nm. These correspond with the reported wavelengths in similar glasses [21,22,23].

The absorption coefficient (*α*) of the examined glasses is computed using the following formula [32,33,34]:

(3) $$\alpha =\frac{1}{d}\ln\left(\frac{I_{0}}{I_{t}}\right)=2.303\frac{OD}{L}$$

where *L* is the thickness of the sample before and after the light traverses the sample, its intensities are *I*_0_ and *I*_t_, and *OD* is the optical density.

**Figure 5:**
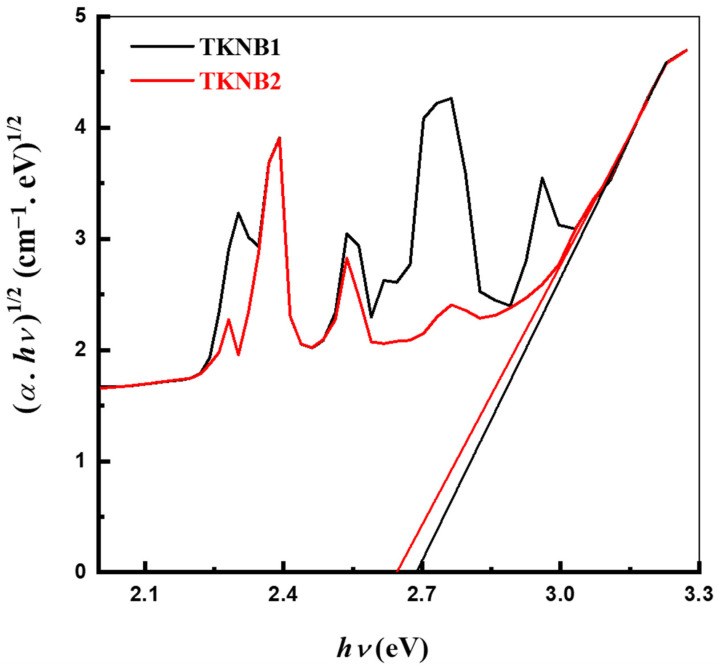
Variation in (*αhν*)^1/2^ versus *hν* for the TKNB glass doped with Ho_2_O_3_/Er_2_O_3_ and Yb_2_O_3_/Er_2_O_3_.

The spectrum of absorption of glasses with varying compositions is used to study changes in the optical band gap (*E*_opt_) and refractive index (*n*). The *E*_opt_, which is the difference between the highest energy in the valence band and the lowest energy in the conduction band, and temperature both affect how many electrons are excited toward the conduction band. When a material’s *E*_opt_ falls within the ranges of 0 to 4 eV, it is classified as a semiconductor. A substance is classified as an insulator if its *E*_opt_ value is substantial, falling between 4 and 12 eV. These statistics are crucial for designing semiconductor devices, since the energy gap determines the electrical and optical characteristics of the device. Clarifying the transformation and electronic band construction of substances has been shown to have been an extremely beneficial effect in research on the optical absorbing edge in the UV area. Using this connection, the optical band gap *E*_opt_ of the specimens is computed from their absorption patterns [35].

(4) $$\alpha \;h\nu =b{\left(h\nu -E_{opt}\right)}^{s}$$

where *b* is a constant, *hv* represents the energy of the incoming photon, and α is the absorbing coefficient. An index called s, which is equivalent to ½, 2, 3/2, or 3 for directly allowed, indirectly allowed, directly forbidden, and indirectly forbidden transitions, respectively, is used to describe the optical absorption processes. In this instance, indirect transmissions in substances are covered by this formula with *s* = 2, which is employed to characterize experimental findings for amorphous substances [33,34,35]. The change in (*αhν*)^1/2^ vs. *hν* for indirect transitions is shown in Figure 5. By means of extrapolation of the linear fitting of the curve in the UV–VIS nm range across the *hν* axis at (*αhν*)^1/2^ = 0, the *E*_opt_ of the fabricated glasses was determined. Table 3 lists each value of *E*_opt_.

It is discovered that the *E*_opt_ values of the two glass samples are lower than the pristine TeO_2_ glass’s 3.79 eV value, determined in a previous study [36]. The values of *E*_opt_ for the TKNB glass co-doped with Er_2_O_3_/Yb_2_O_3_ (TKNB2) are slightly lower than the TKNB glass doped with Er_2_O_3_/Ho_2_O_3_ (TKNB1). The BO in the glass-forming system is altered by the addition of rare earth ions, and any alteration in BO, including the creation of NBO, alters the absorption properties, which in turn reduces the optical band gap. The TeO_2_ matrix’s coordination number varies when rare earth is added. The sample matrix’s structural organization and chemical makeup both have an impact on the optical band gap [37]. When Ho_2_O_3_ is replaced with Yb_2_O_3_, the slight decrease in *E*_opt_ is because of the substitution of the Ho-O bond with the Yb-O bond. The decrease in *E*_opt_ with the addition of Yb_2_O_3_ can be elucidated by the change in electron density [38]. When Yb_2_O_3_ is added to a glass sample instead of Ho_2_O_3_, the amount of NBO may increase, which consequently decreases the *E*_opt_ [37]. The value of the materials’ molar mass is the cause of the increase in NBO [39]. In comparison to the molar mass of Ho_2_O_3_, the molar mass of Yb_2_O_3_ is higher. Thus, there is an increase in NBO atoms, which lowers the value of *E*_opt_ for the Yb_2_O_3_-containing glass sample. Additionally, the *E*_opt_ falls under the category of semiconductor substances, ranging from 2.689 eV to 2.663 eV [40].

**Figure 6:**
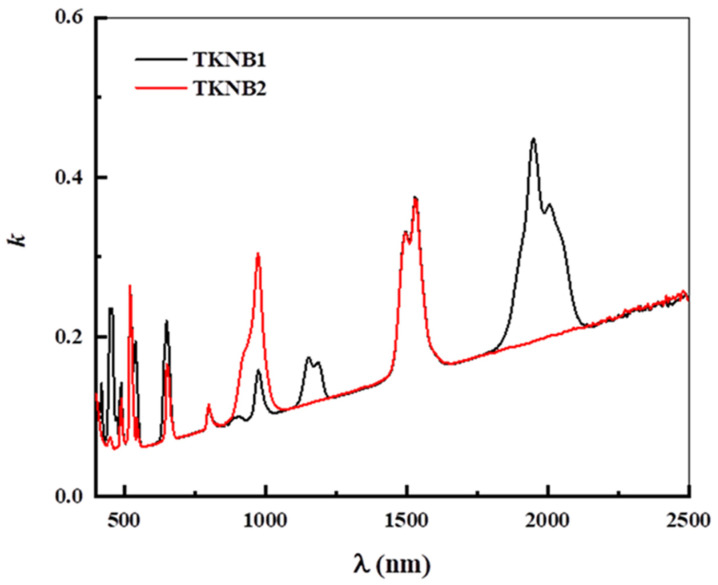
Variation in the extinction coefficient (*k*) with wavelength (*λ*) of the TKNB glass doped with Ho_2_O_3_/Er_2_O_3_ and Yb_2_O_3_/Er_2_O_3_.

**Figure 7:**
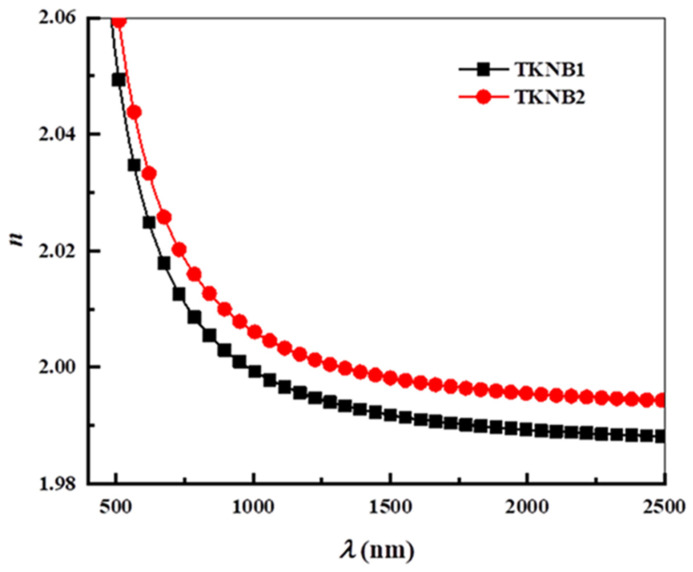
Variation in the refractive index (*n*) with wavelength (*λ*) of the TKNB glass doped with Ho_2_O_3_/Er_2_O_3_ and Yb_2_O_3_/Er_2_O_3_.

The following equations characterize the extinction coefficient (*k*) and *n* of the examined glasses [41]:

(5) $$k=\frac{\alpha \lambda }{4\pi }$$

(6) $$R=\frac{{\left(n-1\right)}^{2}+k^{2}}{{\left(n+1\right)}^{2}+k^{2}};\;n=\left(\frac{1+R}{1-R}\right)+{\left(\frac{4R}{{\left(1-R\right)}^{2}}-k^{2}\right)}^{\frac{1}{2}}$$

where *R* is the reflectance. Figure 6 and Figure 7 show the computed values of the *k* and *n* of the examined glasses, respectively. Both the variations in structure and the fluctuation in the incident wavelength (λ) can affect the *n* and *k*. According to Figure 7, the *n* drops as the λ increases. The following figure shows the various optical factors that are determined using the *n* and *k* spectrum distributions vs. *λ* for the tested glasses.

**Figure 8:**
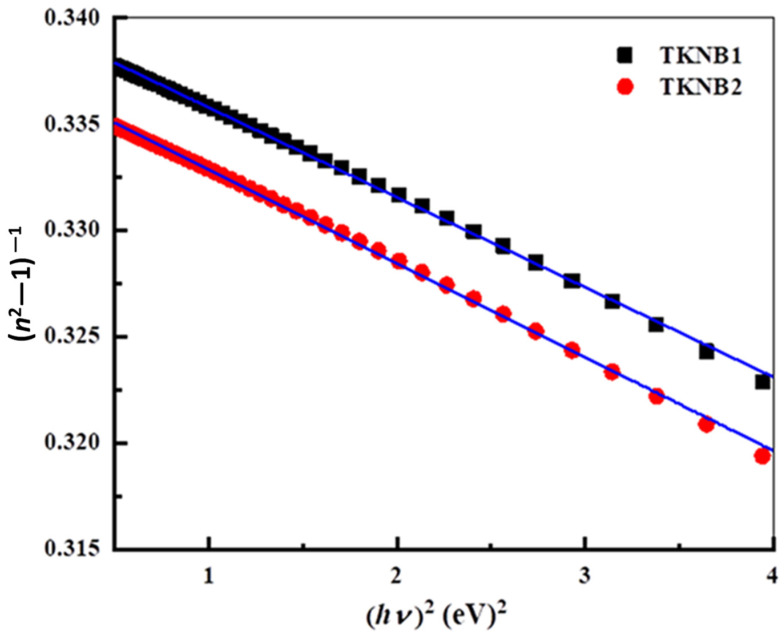
Variation in refractive index (*n*) with wavelength (*λ*) of the TKNB glass doped with Ho_2_O_3_/Er_2_O_3_ and Yb_2_O_3_/Er_2_O_3_.

The data for the refractive indexes in Figure 7 are fitted to a three-term Sellmeier equation:

(7) $$n^{2}\left(\lambda \right)=A+B/\left(1-\frac{C}{\lambda^{2}}\right)+D/\left(1-\frac{E}{\lambda^{2}}\right)$$

where *A*, *B*, *C*, *D*, and *E* are the glass substance dispersion factors (Sellmeier coefficients), and λ is the wavelength in μm. The *n* is influenced by both smaller and greater energy gaps from electronic adsorption, as indicated by the first and second terms. The final term indicates the refractive index-lowering effect of network absorbing [42]. The square of twice the IR transmitting edge could be utilized for calculating the *E* [42,43]. Table 3 displays Sellmeier’s factors, which were obtained by fitting the experimental data [44] with the use of Equation (7). The Wemple and DiDomenico (WDD) single-oscillator model was utilized to study the *n* dispersion [45]. The relation between *n* and *hν* can be represented as follows utilizing this model:

(8) $${\left(n_{0}^{2}-1\right)}^{-1}=\frac{E_{o}}{E_{d}}-\frac{1}{E_{d}E_{o}}{\left(h\nu \right)}^{2}$$

where *E*_d_ is the dispersion energy, which is an estimate of the oscillator strength or average strength of the interband optical transition, and *E*_o_ is the oscillator energy. Equation (8) may be used to derive the *E_o_* and *E_d_* through graphing (*n*^2^−1)^−1^ versus (hv)^2^, as Figure 8 illustrates. Applying the fit of the linear parameters, *E*_o_ and *E*_d_ may be calculated based on the graph. The intercepts and slopes of the arcs yield the Ed and Eo values.

When *hv*→0, the Wemple–DiDomenico dispersion relationship, Equation (8), is extrapolated to obtain the static refractive index (*n*_0_) of the as-prepared glasses, which yields the following formula:

(9) $$n_{0}=\sqrt{1+E_{d}/E_{o}}$$

The determined values of *E*_o_, *E*_d_, and *n*_o_ are recorded in Table 4. It can be seen that the value of *n*_o_ for TKNB glass doped with Er_2_O_3_/Yb_2_O_3_ (TKNB2) is higher than that of TKNB glass doped with Er_2_O_3_/Ho_2_O_3_ (TKNB1) due to the higher density of the TKNB2 sample.

Important factors for the production of optical equipment, such as fiber optic and laser substances, are the *n*, molar polarizability (*α*_m_), and molar refraction (*R*_m_). Consequently, the following formulas [34] are applied to calculate these properties of the examined glasses:

(10) $$R_{m}=\left(\frac{n_{o}^{2}-1}{n_{o}^{2}+2}\right)V_{m}$$

(11) $$\alpha_{m}=\left(\frac{3}{4\pi N_{A}}\right)R_{m}$$

where *N_A_* is Avogadro’s number. The *R_m_* and *α_m_* values are recorded in Table 3. These values for TKNB glass doped with Er_2_O_3_/Yb_2_O_3_ (TKNB2) are higher than those of glass doped with Er_2_O_3_/Ho_2_O_3_ (TKNB1). This provides a qualitative explanation for the rise in refractive indices observed when Yb_2_O_3_ replaces Ho_2_O_3_. We conclude that a rise in molar refraction may be the cause of the observed pattern, which is a rise in the oxide ion polarizability, accompanied by an elevation in *n*. This relationship [31] was utilized to estimate the metallization criterion (*M*) for the as-prepared glasses:

(12) $$M=1-\frac{R_{m}}{V_{m}}$$

The metallic or non-metallic character of the substance is indicated by the *M*. *M* < 0 suggests a metallic nature of the substances, whilst a value of *M* > 0 indicates an insulating nature. The *M* values are listed in Table 3 and were between 0.503 and 0.507. Therefore, the produced glasses demonstrated an insulating nature [30,31]. On the other hand, the exchange of Ho_2_O_3_ with Yb_2_O_3_ causes an upsurge in the valance band’s width and a decrease in *M* and, thus, a decrease in *E*_opt_. As shown in Table 3, the glass doped with Er_2_O_3_/Ho_2_O_3_ had higher values of *M* and *E*_opt_, while the glass co-doped with Er_2_O_3_/Ho_2_O had smaller values of *M* and *E*_opt_.

The nonlinear optical parameters can be determined based on Miller’s rule. The dispersion of the optical linear susceptibility *χ*^(1)^ and the third-order nonlinear optical susceptibility *χ*_(3)_ are, respectively, deduced based on their empirical relations [46].

(13) $$\chi^{\left(1\right)}=\frac{n_{o}^{2}-1}{4\pi }$$

(14) $$\chi^{\left(3\right)}={\left[\chi^{\left(1\right)}\right]}^{4}\times 1.7\times 10^{-10}$$

The nonlinear refractive index n_2_ of the examined glasses relates to the third-order nonlinear optics *χ*^(3)^ and static refractive index *n*_o_, which can be determined using the following equation [47,48]:

(15) $$n_{2}=\frac{12\pi }{n_{o}}\chi^{\left(3\right)}$$

The calculated values of *χ*^(1)^, *χ*^(3)^, and *n*_2_ are listed in Table 4. It can be observed that these values are higher for the TKNB glass doped with Yb_2_O_3_/Er_2_O_3_ (TKNB2) than for the TKNB glass doped with Ho_2_O_3_/Er_2_O_3_ (TKNB1). The reported results demonstrate an increase in the nonlinear susceptibility, and the examined glasses show high values of optical linear susceptibility, *χ^(^*^1)^, replacing an atom with an atomic radius that is smaller than the removed one. This also applies when replacing Ho^3+^ (atomic radii = 2.33 Å) with Yb^3+^ (atomic radius = 2.28 Å) in the TKNB glass doped with Yb_2_O_3_/Er_2_O_3_ (TKNB2), which causes an increase in the *χ*^(3)^ value. This is dependent on the value of the third-order nonlinear optical susceptibility, *χ*^(3)^, and nonlinear refractive index, n_2_, which suggest that these glasses can be used in nonlinear optical devices, especially in the communication bands.

### 3.4. Absorption Spectra and Judd and Ofelt Analysis

**Figure 9:**
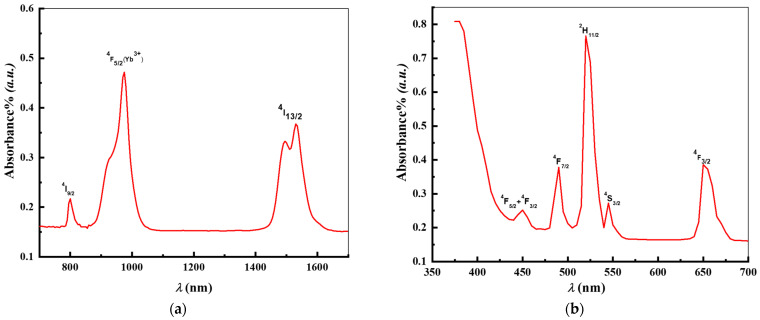
RT absorption spectrum of Er^3+^/Yb^3+^-co-doped tellurite glass (TKNB2): (**a**) near-infrared range and (**b**) UV–visible range.

Figure 9 displays the Er^3+^/Yb^3+^-co-doped glasses and their absorption spectra in the UV–VIS–NIR wavelength between 400 and 1800 nm. The 4f^11^-4f^11^ transitions of Er^3+^ and Yb^3+^ are responsible for all of the bands. Three bands at 800, 975, and 1530 nm are detectable in the infrared section of the spectrum (Figure 9a). The first and third bands result from Er^3+^ transitions between ^4^I_9/2_ and ^4^I_13/2_, which are ground states, and ^4^I_15/2_. With an increased optical density, the second band represents an overlapping absorption among the two transmissions, ^2^F_7/2_→^2^F_5/2_ (originating from Yb^3+^ ion) and ^4^I_15/2_→^4^I_11/2_ (originating from Er^3+^ ion). As seen in Figure 9b, the UV–visible region of the absorbed spectrum demonstrates well-resolved lines, which are ascribed to the transition of Er^3+^ ions in the ground state, ^4^*I*_15/2_ to the ^4^*F*_3/2_, ^4^*F*_3/2_, ^4^*F*_5/2_, ^4^*F*_7/2_, ^4^*H*_11/2_, ^4^*S*_3/2_, and ^4^*F*_9/2_. These absorption bands were assigned and located in accordance with Carnall et al. [49] and all relevant research.

The absorption spectra of this glass medium (TKNB2) were subjected to a Judd and Ofelt (JO) investigation in order to ascertain their spectroscopic characteristics. Below is a quick synopsis of the JO investigation.

The well-known theory developed by JO in 1962 makes it possible to compute the probability of an electric dipole transition between rare earth ion energy levels in diverse contexts [50]. Theoretically, the electric $S_{ed}^{cal}$ and magnetic ($S_{md}$) dipole line strengths can be utilized for describing the radiative transmissions of the first J level to the succeeding J′ level in the 4fn conformation of rare earth ions [51,52].

The calculated line strength $S_{ed}^{calc}\left(SLJ,S^{\prime }L^{\prime }J^{\prime }\right)$ between the initial state J, described by $\left(S,L,J\right),$ and the final state $J^{\prime },$ given by ($S^{\prime },L^{\prime },J^{\prime }),$ can be expressed using the following relation [53,54]:

(16) $$S_{ed}^{calc}\left(SLJ,S^{\prime }L^{\prime }J^{\prime }\right)=\sum_{t=2,4,6}\Omega_{t}{\left|\langle SLJ\left\|U^{\left(t\right)}\right\|S^{\prime }L^{\prime }J^{\prime }\rangle \right|}^{2}$$

The influence of the host glasses on the luminescence intensity is represented by the JO intensity factors, $\Omega_{t}\;(t=2,\;4,\;6)$, and the doubly reduced matrix elements of grade t among the statuses with the quantum numbers of (S,L,J) and $\left(S^{\prime },L^{\prime },J^{\prime }\right)$ are ${\left|\langle SLJ\left\|U^{\left(t\right)}\right\|S^{\prime }L^{\prime }J^{\prime }\rangle \right|}^{2}$ [51]. The reduced matrix elements, which may be obtained in the literature [52,53,54,55,56,57,58], are only dependent on the angular momentum of the Er^3+^ states. The reduced matrices are defined as the total of the relevant matrix elements for two or more manifolds. Table 5 provides the matrix element values for each Er^3+^ absorption band.

Meanwhile, the measured electric dipole line strength $S_{ed}^{meas}\left(SLJ,S^{\prime }L^{\prime }J^{\prime }\right)$ can also be calculated from the absorption spectra (Figure 1) [56,57,58] by using the following equation:

(17) $$S_{ed}^{meas}=\left[\frac{9n}{{\left(n^{2}+2\right)}^{2}}4\pi \epsilon_{0}\frac{3ch\left(2J+1\right)}{8\pi^{3}e^{2}}\times \frac{2.303}{LN\bar{\lambda }}\int_{J\to J^{\prime }}^{j}OD\left(\lambda \right)d\lambda \right]-n\cdot S_{md}\left(SLJ,SLJ^{\prime }\right)$$

where *J* is the total angular momentum quantum number of the ground state J=152, *e* is the charge of the electron, and *N* is the ion content (ions/cm^3^). The average wavenumber of the absorbing band is denoted by $\bar{\lambda }\;(\mathrm{nm})$, the refractive index of the host with respect to $\bar{\lambda }$ is represented by *n*, *L* is the thickness of the sample under study (*L* = 1 mm), and $\int_{J\to J^{\prime }}^{j}OD\left(\lambda \right)d\lambda$ symbolizes the experimentally determined integrated optical density in the respective wavelength ranges.

The magnetic dipole transitions $S_{md}\left(SLJ,SLJ^{\prime }\right)$ contribute as follows [59]:

(18) $$S_{md}\left(SLJ,SLJ^{\prime }\right)={\left(\frac{h}{4\pi mc}\right)}^{2}{\left|\langle SLJ\left\|L+2S\right\|SLJ^{\prime }\rangle \right|}^{2}$$

The symbols m and c stand for the electron mass and light velocity, respectively. The magnetic dipole matrix elements between the LS-coupled states are represented by $\langle S\left\|L+2S\right\|SLJ^{\prime }\rangle$ [60].

Table 6 demonstrates that the only transition with a contribution of the magnetic dipole, $S_{md}=0.7148\times 10^{-20}\;{cm}^{2}$, in the case of Er^3+^ ions is the ^4^*I*_15/2_*→*^4^*I*_13/2_ (selection rules: ∆J=1).

The measured line strengths $(S_{ed}^{meas}$) of the electric dipoles were utilized to calculate the values of the JO intensity factors $\Omega_{2},\Omega_{3},\;and\;\Omega_{6}$. If the JO factors produce a column vector Ω, and the double-decreased matrix elements produce an n×3 matrix A, where n is the number of transmissions to fit and 3 matches the three JO factors, then the measured electric dipole line strength can be transcribed as a 1×n column vector. The equality among Equations (13) and (14) can be articulated as $S_{ed}^{meas}=A.\Omega$. The group of JO factors was obtained from the matrix $\Omega ={\left(A^{T}.A\right)}^{-1}.A^{T}.S_{ed}^{meas}$, where $A^{T}$ is the transposition of matrix A. This matrix-based process is perfect for computations that are carried out on a computer.

The optimal modification was performed, accounting for the initial six transitions and yielding the following values: $\Omega_{2}=2.387\times 10^{-20}\;{cm}^{2}$, $\Omega_{4}=1.881\times 10^{-20}\;{cm}^{2}$, and $\Omega_{6}=0.657\times 10^{-20}\;{cm}^{2}$. These computed $\Omega_{t}$ values were utilized in Equation (12) to obtain the values of $S_{ed}^{calc}$. Table 6 provides an overview of the outcomes of some of the calculated parameters, as well as the $S_{ed}^{calc}$ and $S_{ed}^{meas}$ absorption line strengths for the Er^3+^/Yb^3+^-co-doped TKNB2 sample.

Table 6 presents the values of some of the factors that were employed in computation, as well as the $S_{ed}^{meas}$ and $S_{ed}^{calc}$ absorbing line strengths for the Er^3+^/Yb^3+^-co-doped TKNB2 sample.

The formula below indicates the root mean square $\delta_{rms}$ difference between the predicted and observed line strengths of the transitions, which serves as a gauge for the fit’s accuracy:

(19) $$\delta_{rms}={\left(\sum_{i=1}^{N_{trans}}\frac{\left(S_{ed}^{meas}(i)-S_{ed}^{cal}(i)\right)}{\left(N_{trans}-3\right)}\right)}^{1/2}$$

where $N_{trans}$ is the number of strong absorption transitions.

The value found in this study, $\delta_{rms}=0.0361\times 10^{-20}\;{cm}^{2}$, is tiny when compared with values found in other kinds of glasses [61,62,63,64,65,66].

The calculated $\Omega_{t}$ parameters for different glasses accord well with those reported in existing works. The $\Omega_{t}$ measurements of Er^3+^ ions in several other common glasses [67,68,69,70,71,72,73] are shown in Table 7. The intensity parameters comprise two terms, based on the JO theory [74]. Firstly, the symmetry and distortions associated with the constructional alteration in the presence of rare earth ions are described by the crystal field parameter. The other represents the covalency between the ligand anions and doped rare earth ions, which is connected to the excited opposite parity electronic states and 4f radial integral states of wave functions. Further, the covalent bonding of binder anions and rare earth ions (less ionic in nature) in the host and the symmetry of the immediate environment surrounding them are correlated with the intensity parameter $\Omega_{2}$. The degree of asymmetry surrounding rare earth sites increases with an increasing value of $\Omega_{2}$, indicating a greater covalency between the metal and ligand link. The bulk characteristics of the glass framework, such as stiffness, viscosity, and basicity, are primarily described by the intensity factors $\Omega_{4}$ and $\Omega_{6}$, which are also influenced by the acidity and alkalinity of the host substance [75]. The host material’s hardness and basicity increase and decrease, respectively, with higher values of $\Omega_{4}$ and $\Omega_{6}$. Table 7 demonstrates that all three intensity parameters exhibit an upward trend of $\Omega_{2}$ > $\Omega_{4}$ > $\Omega_{6}$, which aligns with the majority of results from previous studies. A greater asymmetry and superior covalence are indicated by the higher $\Omega_{2}$ value compared with $\Omega_{4}$ and $\Omega_{6}$ [76]. In the meantime, all three Er^3+^ intensity characteristics that were measured in the current study were reduced to within the values of other glass hosts, suggesting a local asymmetry associated with the Er^3+^/Yb^3+^ ions and moderate covalency of the Er-O/Yb-O bonds in the current TeO_2_ glass. The manufactured glass’s spectroscopic quality factor, $Q=\Omega_{4}/\Omega_{6}$, has a value of 2.8630. When compared with the glass hosts ZBLAN [77], Boro-tellurite [78], and TLNT [79], the generated glass had a higher value of $Q=\Omega_{4}/\Omega_{6}$. The glass that was developed had higher spectroscopic quality factor values, $Q=\Omega_{4}/\Omega_{6}$, than those found for TeO_2_ glasses in earlier investigations, indicating that it is a better fit for optical devices. For optical amplifier and laser usage, the glass used in the present research (TKNB2) is a great option [80].

The relationship between the emission line strengths related to the transmissions from the highest energy levels, ^4^*I*_15/2_, ^4^*I*_13/2_, ^4^*I*_11/2_, ^4^*I*_9/2_, ^4^*F*_9/2_, ^4^*S*_3/2_, ^2^*H*_11/2_, and ^4^*F*_7/2_, and their related lower-lying energy levels can be computed utilizing the JO intensity parameters. The spontaneous emission probabilities $A_{rad}(J\to J^{\prime })$ can be calculated utilizing these line strengths as follows:

(20) $$A_{rad}\left(J\to J^{\prime }\right)=A_{ed}+A_{md}=\frac{e^{2}}{4\pi \epsilon_{0}}\frac{64\pi^{4}}{3h\left(2J+1\right){\bar{\lambda }}^{4}}\left(\frac{{n\left(n^{2}+2\right)}^{2}}{9}S_{ed}+n^{3}S_{md}\right)$$

where $A_{ed}$ and $A_{md}$ denote the respective radiative transmission probabilities for electric and magnetic dipoles. Equations (13) and (15) are employed to calculate $S_{ed}$ and $S_{md}$, respectively.

The relative transmission probability, or branching ratio $\beta_{rad}\left(J\to J^{\prime }\right)$, can be obtained using the following equation if there are many transitions from the level:

(21) $$\beta_{rad}\left(J\to J^{\prime }\right)=\frac{A_{rad}\left(J\to J^{\prime }\right)}{\sum A_{rad}\left(J\to J^{\prime }\right)}$$

where all terminal states *J*′ are covered by the total.

An energy level’s radiative lifetime $\tau_{rad}$, which is based on the rates of spontaneous emission across all transitions from this level, can be calculated utilizing the following equation:

(22) $$\tau_{rad}=\frac{1}{\sum A_{rad}\left(J\to J^{\prime }\right)}$$

Table 8 presents the electric dipole transmission probability $A_{ed}$, magnetic dipole transmission probability $A_{md}$, spontaneous emission transmission probability $A_{rad}$, fluorescence branch ratio $\beta_{rad}$, and radiation lifetime $\tau_{rad}$ of the Er^3+^ ions in TKNB2 glass, which relate to the transmissions that occur from the higher energy levels of ^4^*I*_13/2_, ^4^*I*_11/2_, ^4^*I*_9/2_, ^4^*F*_9/2_, ^4^*S*_3/2_, ^4^*H*_11/2_, and $F_{7/2}$ to their lower-lying energy levels, respectively.

**Figure 10:**
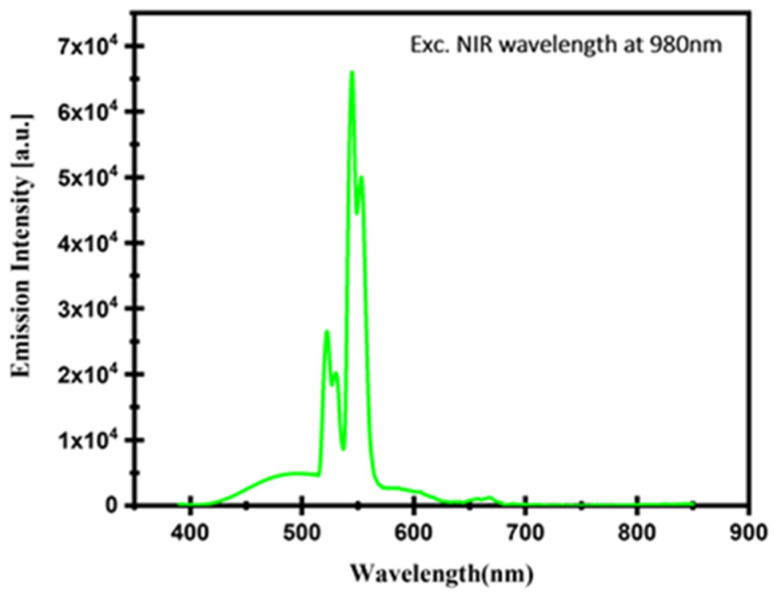
Emission spectra of Er^3+^/Yb^3+^-co-doped glass at excitation wavelength of 980 nm.

In general, the green and red UV emissions were below NIR excitement for the Er^3+^ and Yb^3+^-co-doped glass (see Figure 10). According to estimates, the branched ratios of the transitions ^2^*H*_11/2_→^4^*I*_15/2_ (green), ^4^*S*_3/2_→^4^*I*_15/2_ (green), and ^4^*F*_9/2_→^4^*I*_15/2_ (red) were 94.8585%, 69.0467%, and 92.5887%, respectively. These findings indicate that the green and red emission transition channels predominate over all related higher-level radiative transitions.

The ions’ capacity for absorbing or emitting light is measured using the absorption and emission cross-sections. A significant emission cross-section indicates a large gain coefficient and lower pump laser threshold energy. Utilizing the Beer–Lambert equation, the absorbing cross-section $\left(\sigma_{a}\right)$ of the ^4^*I*_13/2_→^4^*I*_15/2_ transmission (1540 nm) of Er^3+^ was found based on the absorbing patterns of Er^3+^/Yb^3+^-co-doped TeO_2_ glass [41]:

(23) $$\sigma_{a}\left(\lambda \right)=\frac{2.303\times OD(\lambda )}{N\times L}$$

where *N* is the content of Er^3+^ ions (ions/cm^3^), *L* is the thickness of the sample, and OD(λ) is the optical density of the realistic absorbing patterns of the manufactured glass. The McCumber theory allows for the extraction of the stimulated emission cross-section ($\sigma_{e})$ utilizing the following relationship [42,43,44]:

(24) $$\sigma_{e}\left(\lambda \right)=\sigma_{a}\left(\lambda \right)\frac{Z_{l}}{Z_{u}}exp\left[\frac{hc}{k_{B}T}\left(\frac{1}{\lambda_{ZL}}-\frac{1}{\lambda }\right)\right]$$

**Figure 11:**
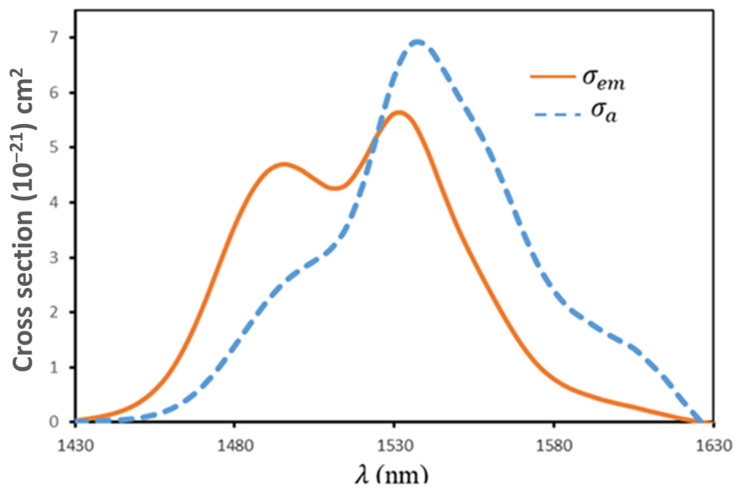
The computed absorption and emission cross-sections of Er^3+^/Yb^3+^-co-doped TeO_2_ glass (TKNB2) for the transition of ^4^*I*_3/2_→^4^*I*_5/2_.

The absorption cross-section and partition functions of the lesser and higher states engaged in the optical transmission under consideration are represented by $\sigma_{a}\left(\lambda \right)$, $Z_{l}$, and $Z_{u}$. The Planck constant $6.63\times 10^{-34}\;J\;S$, Boltzmann constant $(1.38\times 10^{-23}$, and temperature (RT in this case) are denoted by the parameters h, $k_{B}$, and T. Additionally, the wavelength at which the Stark sublevels of the emission are lower and multiple transmissions are achieved is denoted by $\lambda_{ZL}$. Figure 11 displays the computed values of the absorbing and emission cross-sections of Er^3+^/Yb^3+^-co-doped TeO_2_ glass (between 1430 and 1630).

The stimulation-emitted cross-section peak, $\sigma_{e}^{peak}$, is located at $6.86\times 10^{-21\;}{cm}^{2}$. This value is in good agreement with those of other TeO_2_ glasses reported in the literature [77,78,79,80]. Moreover, the emitted cross-section $\sigma_{e}^{peak}$ and the full width at half-maximum (FWHM) are crucial factors in obtaining high gain and wideband amplification in optical amplifiers. The resulting $FWHM\times \sigma_{e}^{peak}$ could be employed to determine an optical amplifier’s bandwidth characteristics. A broader gain bandwidth and lower pump threshold power are implied by the higher values of the $FWHM\times \sigma_{e}^{peak}$ produced and the higher radiative lifetime $\tau_{rad}$. Table 9 lists the $FWHM\times \sigma_{e}^{peak}$ values of Er^3+^/Yb^3+^-co-doped glasses. It can be seen that the $FWHM\times \sigma_{e}^{peak}$ in TKNB2 has a comparable bandwidth characteristic to other glass hosts such as phosphate tellurite glass ($337.5\times 10^{-21}\;nm\cdot cm^{2})$ and PBGG $\left(338.5\times 10^{-21}\;nm\cdot cm^{2}\right)$ [35]. Finally, we found that the effect of Er^3+^/Ho^3+^ was slightly changed compared with Er^3+^/Yb^3+^ in the same host glass; therefore, we only reported the emission cross-section and increased spectroscopic values for a transition energy level of ^4^*I*_3/2_→^4^*I*_5/2_ of TKNB2.

When assessing the effectiveness of laser mediums, the optical gain coefficient is an essential factor to consider. Once the absorbed and emitted cross-sections for the changes between the two working laser levels are known, the following equation can be applied to determine the optical gain coefficient G(λ) [41]:

(25) $$G\left(\lambda \right)=N\left[P\sigma_{e}\left(\lambda \right)-(1-P)\sigma_{a}\left(\lambda \right)\right]$$

where P is the rate of population inversion.

**Figure 12:**
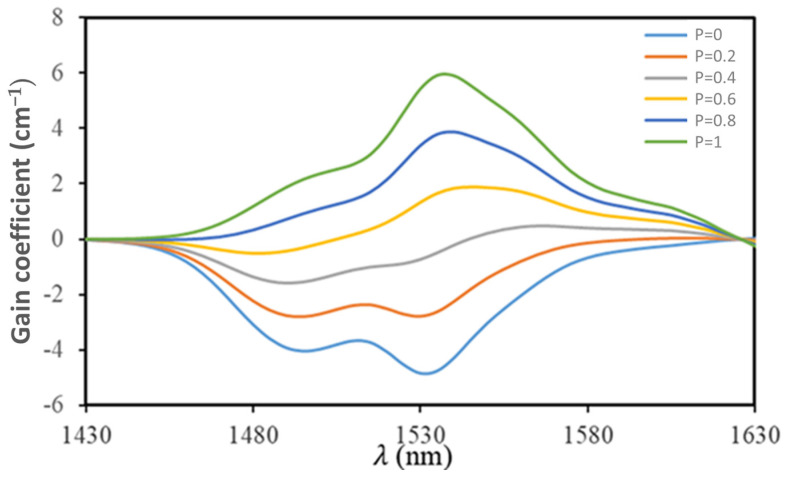
The gain coefficient for the ^4^*I*_3/2_→^4^*I*_5/2_ transition of Er^3+/^Yb^3+^-co-doped tellurite glass (TKNB2).

The electron population ratio across the two energy levels is represented by the *p* value, which progressively increases from 0 to 1, as seen in Figure 12. This figure demonstrates that a positive gain can be achieved when *p* ≥ 0.6, suggesting that TKNB2 glass may find a use as a matrix substance for 1.54 μm fiber lasers.

## 4. Conclusions

The thermal stability parameters for the Er_2_O_3_/Yb_2_O_3_-co-doped glass were slightly lower than those for Er_2_O_3_/Ho_2_O_3_ due to the substitution of Ho_2_O_3_ with Yb_2_O_3_ in the glassy matrix. The value of *E*_opt_ reduced from 2.689 to 2.663 eV following the substitution of Ho_2_O_3_ with Yb_2_O_3_, because this substitution increased the amount of NBO in the glass network. The physical parameters (*α*_m_, *R*_m_, *E*_d_, *E*_o,_ and *n*_o_) were correlated to the existence of rare earth oxides (Er_2_O_3_/Ho_2_O_3_ and Er_2_O_3_/Yb_2_O_3_) in the glassy matrix. The spectroscopic characteristics of the Er^3+^/Yb^3+^-co-doped glasses were evaluated based on their intensity factors, radiative rates, branching ratios, and radiative lifetimes. The maximum emission cross-section reported was $6.8\times 10^{-21}\;{cm}^{2}$, and the gain coefficient of Er^3+^/Yb^3+^ for the transition of ^4^*I*_13/2_→^4^*I*_15/2_ was 6.0 cm^−1^ with a high quality factor ($\sigma_{e}\times FWHM=377.3\times 10^{-21}\;nm\cdot cm^{2})$. When the Ho^3+^ ions are replaced with Yb^3+^ ions in the same host glass, it leads to a slight change in the spectroscopic properties, viz, the emission cross-section and gain and intensity parameters of the transition energy level, ^4^*I*_13/2_→^4^*I*_15/2_. These findings suggest that the co-doping of tellurite glass is a viable choice for optical amplifier and laser manufacturing in the communication bands.

## Figures and Tables

**Table 1:** The composition, density *ρ*, and molar volume *V*_m_ of the TKNB glassy system co-doped with Er_2_O_3_/Ho_2_O_3_ and Er_2_O_3_/Yb_2_O_3_.

Sample Code	Composition (mol%)	*Ρ* (g/cm^3^) ± 0.001	*V*_m_ (cm^3^/mol) ± 0.0056
TKNB1	70TeO_2_-15K_2_TeO_3_-10Nb_2_O_5_-5BaF_2_-30,000 ppm Ho_2_O_3_-30,000 ppm Er_2_O_3_	4.9585	35.373
TKNB2	70TeO_2_-15K_2_TeO_3_-10Nb_2_O_5_-5BaF_2_-30,000 ppm Yb_2_O_3_-30,000 ppm Er_2_O_3_	4.9622	35.353

**Table 2:** Thermal parameters of the TKNB glass system co-doped with Er_2_O_3_/Ho_2_O_3_ and Er_2_O_3_/Yb_2_O_3_.

Sample Code	*T*_g_ (°C) ± 1.0	*T*_c_ (°C) ± 0.1	*T*_p_ (°C) ± 1.0	∆*T* (°C) ± 1.0	*H* ± 1.0	*K_SP_* ± 1.0
TKNB1	333	422	447	89	0.267	8.56
TKNB2	332	419	442	87	0.262	7.62

**Table 3:** The Sellmeier coefficients, molar refractivity *R*_m_, molar polarizability *α*_m_, metallization criterion *M*, and optical band gap *E*_opt_ of the TKNB glass system co-doped with Er_2_O_3_/Ho_2_O_3_ and Er_2_O_3_/Yb_2_O_3_.

Sellmeier Coefficients					*R*_m_ (Mol^−1^) ± 0.0465	*α*_m_ (Å^−3^) ± 0.0064	*M* ± 0.0008	*E*_opt_ (eV) ± 0.001
Sample Code	A	B	C	D				
TKNB1	3.195	0.7393	0.2552	0.01397	17.449	6.921	0.507	2.689
TKNB2	3.205	0.7512	0.2593	0.01695	17.558	6.964	0.503	2.663

**Table 4:** Dispersion and nonlinear optical parameters of the TKNB glass system co-doped with Ho_2_O_3_/Er_2_O_3_ and Yb_2_O_3_/Er_2_O_3_.

Sample Code	Dispersion Parameters			Nonlinearity Parameters		
	*E*_o_ (eV)	*E*_d_ (eV)	*n*_o_ ± 0.001	*χ* ^(1)^	*χ*^(3)^ × 10^−13^ *(esu)*	*n*_2_ × 10^−12^ *(esu)*
TKNB1	8.59	25.28	1.98	0.23407	5.1	9.68
TKNB2	8.75	25.95	1.99	0.23596	5.26	9.97

**Table 5:** Reduced matrix element values for Er^3+^ absorption transitions.

Transition from ^4^*I*_15/2_ to	${\left\|U^{\left(2\right)}\right\|}^{2}$	${\left\|U^{\left(4\right)}\right\|}^{2}$	${\left\|U^{\left(6\right)}\right\|}^{2}$
^4^ *I*_13/2_	0.0194	0.1173	1.4332
^4^ *I*_9/2_	0.0	0.1694	0.0101
^4^ *F*_9/2_	0.0	0.548	0.4769
^4^ *S*_3/2_	0.0	0.0	0.2369
^2^ *H*_11/2_	0.8120	0.4669	0.0951
^4^ *F*_7/2_	0.0	0.1447	0.6348

**Table 6:** Values of average wavelengths, refractive index, and integrated absorption coefficients’ electric and magnetic dipole line strengths for Er^3+^/Yb^3+^-co-doped tellurite glass (TNBK2).

Transition from ^4^*I*_15/2_ to	$\bar{\lambda }$ (nm)	n	$\int OD\left(\lambda \right)d\lambda$ (nm)	$S_{ed}^{cal}$ $\left(pm^{2}\right)$	$S_{ed}^{mes}$ $\left(pm^{2}\right)$	$S_{md}$ $\left(pm^{2}\right)$
^4^ *I*_13/2_	1530	1.9975	17.88414	1.2087	1.1855	0.7148
^4^ *I*_9/2_	800	2.0155	1.06715	0.3254	0.2938	0
^4^ *F*_9/2_	650	2.0286	4.02307	1.3446	1.3483	0
^4^ *S*_3/2_	545	2.0480	0.46898	0.1558	0.1845	0
^2^ *H*_11/2_	520	2.0554	7.03041	2.8796	2.8801	0
^4^ *F*_7/2_	490	2.0669	1.70483	0.6949	0.7341	0
				$\delta_{rms}=0.0361\times 10^{-20}\;{cm}^{2}$		

**Table 7:** Judd–Ofelt parameters and spectroscopic quality factor ($Q=\Omega_{4}/\Omega_{6}$) in different host materials, along with reported Er^3+^-doped systems.

Glass Host	$\Omega_{2}\times 10^{-20}\;{cm}^{2}$	$\Omega_{4}\times 10^{-20}\;{cm}^{2}$	$\Omega_{6}\times 10^{-20}\;{cm}^{2}$	$Q=\Omega_{4}/\Omega_{6}$	Reference
Er^3+^/Yb^3+^	2.387	1.881	0.657	2.8630	[Present work]
ZBLAN	2.73	1.40	1.10	1.2727	[77,80]
Boro-tellurite	4.232	0.779	0.612	1.2729	[78,81]
Tellurite	5.6	2.11	0.73	2.8904	[79,82]
TLNT	6.48	1.82	1.27	1.4331	[80,83]

**Table 8:** Calculated radiative parameters of the different states of Er^3+^ ion-doped tellurite glass (TKNB2).

Transitions	$\bar{\nu }({cm}^{-1})$	n	$S_{ed}\;(pm^{2})$	$S_{md}\;(pm^{2})$	$A_{ed}\;(s^{-1})$	$A_{md}\;(s^{-1})$	β(%)=	$\tau_{r}(ms)$
^4^*I*_13/2_→^4^*I*_15/2_	6536	1.998	1.2079	0.7000	277	80.4906	100.00	2.7956
^4^*I*_11/2_→^4^*I*_15/2_	10,256	2.007	0.3205	0	339	0	89.617	2.6416
^4^*I*_11/2_→^4^*I*_13/2_	3720	1.995	0.4040	0.7910	20	19.4855	10.383	
^4^*I*_9/2_→^4^*I*_15/2_	12,500	2.016	0.3253	0	762	0	85.548	1.1221
^4^*I*_9/2_→^4^*I*_13/2_	5964	1.997	0.5063	0	123	0	13.838	
^4^*I*_9/2_→^4^*I*_11/2_	2244	1.995	0.2490	0.3480	3	2.2576	0.6143	
^4^*F*_9/2_→^4^*I*_15/2_	15,385	2.029	1.3441	0	605.5	0	92.589	0.1529
^4^*F*_9/2_→^4^*I*_13/2_	8849	2.003	0.3787	0	306	0	4.6771	
^4^*F*_9/2_→^4^*I*_11/2_	5129	1.996	1.0018	0.1720	155	13.3420	2.5720	
^4^*F*_9/2_→^4^*I*_9/2_	2885	1.995	0.3303	0.1120	9	1.5441	0.1623	
^4^*S*_3/2_→^4^*I*_15/2_	18,349	2.048	0.1556	0	310.9	0	69.047	0.2221
^4^*S*_3/2_→^4^*I*_13/2_	11,813	2.013	0.2298	0	113.0	0	25.086	
^4^*S*_3/2_→^4^*I*_11/2_	8093	2.001	0.0646	0	99	0	2.2064	
^4^*S*_3/2_→^4^*I*_9/2_	5849	1.996	0.2848	0	164	0	3.6314	
^4^*S*_3/2_→^4^*F*_9/2_	2964	1.995	0.0181	0	1	0	0.0299	
^2^*H*_3/2_→^4^*I*_15/2_	19,231	2.055	2.8790	0	2245.2	0	94.859	0.0423
^2^*H*_3/2_→^4^*I*_13/2_	12,695	2.016	0.2473	0.1160	507	117.2763	2.6367	
^2^*H*_3/2_→^4^*I*_11/2_	8975	2.003	0.3695	0.0860	260	30.1353	1.2244	
^2^*H*_3/2_→^4^*I*_9/2_	6731	1.998	0.8182	0.0300	239	4.3980	1.0303	
^2^*H*_11/2_→^4^*F*_9/2_	3846	1.995	1.0819	0.0090	59	0.2450	0.2489	
^2^*H*_11/2_→^4^*S*_3/2_	882	1.995	0.4219	0	0	0	0.0012	
^4^*F*_7/2_→^4^*I*_15/2_	20,408	2.067	0.6945	0	996.8	0	71.627	0.0719
^4^*F*_7/2_→^4^*I*_13/2_	13,872	2.021	0.6294	0	255.4	0	18.355	
^4^*F*_7/2_→^4^*I*_11/2_	10,152	2.0070	0.6296	0	969	0	6.9622	
^4^*F*_7/2_→^4^*I*_9/2_	7908	2.0005	0.5105	0.051	366	18.2576	2.7589	
^4^*F*_7/2_→^4^*F*_9/2_	5023	1.9955	0.1131	0.212	21	19.3050	0.2862	
^4^*F*_7/2_→^4^*S*_3/2_	2059	1.9947	0.0087	0	0	0	0.0008	
^4^*F*_7/2_→^4^*H*_11/2_	1177	1.9947	0.6207	0	1	0	0.0104	

**Table 9:** Obtained values of FWHM (nm), $\sigma_{e}\;(cm^{2})$ 1.54 μm, and $\sigma_{e}\times FWHM\;(nm\cdot cm^{2})$ at 1.53 μm.

	FWHM (nm)	$\sigma_{e}\;(cm^{2})$	$\sigma_{e}\times FWHM\;(nm\cdot cm^{2})$
Present work Er^3+^/Yb^3+^	55	6.86 $\times \;10^{-21}\;cm^{2}$	$377.3\times 10^{-21}\;nm\cdot cm^{2}$
PBGG [35]	38	8.9	$338.5\times 10^{-21}\;nm\cdot cm^{2}$
Phosphate tellurite glass [36]	44.842	7.7	$337.5\times 10^{-20}\;nm\cdot cm^{2}$

## Data Availability

The original contributions presented in the study are included in the article, further inquiries can be directed to the corresponding author.
